# Coming Back Around: Lessons From the Second Cycle of Age Friendly Boston Planning

**DOI:** 10.1093/geroni/igaf122.1199

**Published:** 2025-12-31

**Authors:** Megan Siebecker, Caitlin Coyle

**Affiliations:** University of Massachusetts Boston, Boston, Massachusetts, United States; University of Massachusetts Boston, Boston, Massachusetts, United States

## Abstract

This presentation demonstrates the evolution of community reengagement in Boston through the second iteration of the Age-Friendly Boston planning process. Boston became a member of the World Health Organization’s Age-Friendly Cities network in 2014, and its first Age-Friendly Boston Action Plan was published in 2017. Through a combination of community forums, surveys, and other community engagement efforts, this presentation highlights key shifts in the needs and priorities of older residents over time with evidence from the 2025 Age-Friendly planning process. Results from a mixed methods analysis of 35 community forums, 1,308 surveys representing 11 languages across the 21 unique neighborhoods in Boston will be presented. Notably, data suggests the shifting needs of Boston’s older adults over time reflects the success of the City’s efforts in instituting the 2017 action plan. While earlier conversations focused primarily on issues like housing and economic security, more recent discussions have increasingly centered on social connection, a sense of belonging, and civic engagement. The evolving needs of the community reflect a growing recognition of the importance of social fulfillment and communication. Additionally, new insights suggest that neighborhood-level differences have emerged as a critical focus. This presentation will highlight the progress made in Age-Friendly Boston’s efforts, while also offering insights into the changing needs and expectations of older Bostonians as they navigate a changing urban environment.

